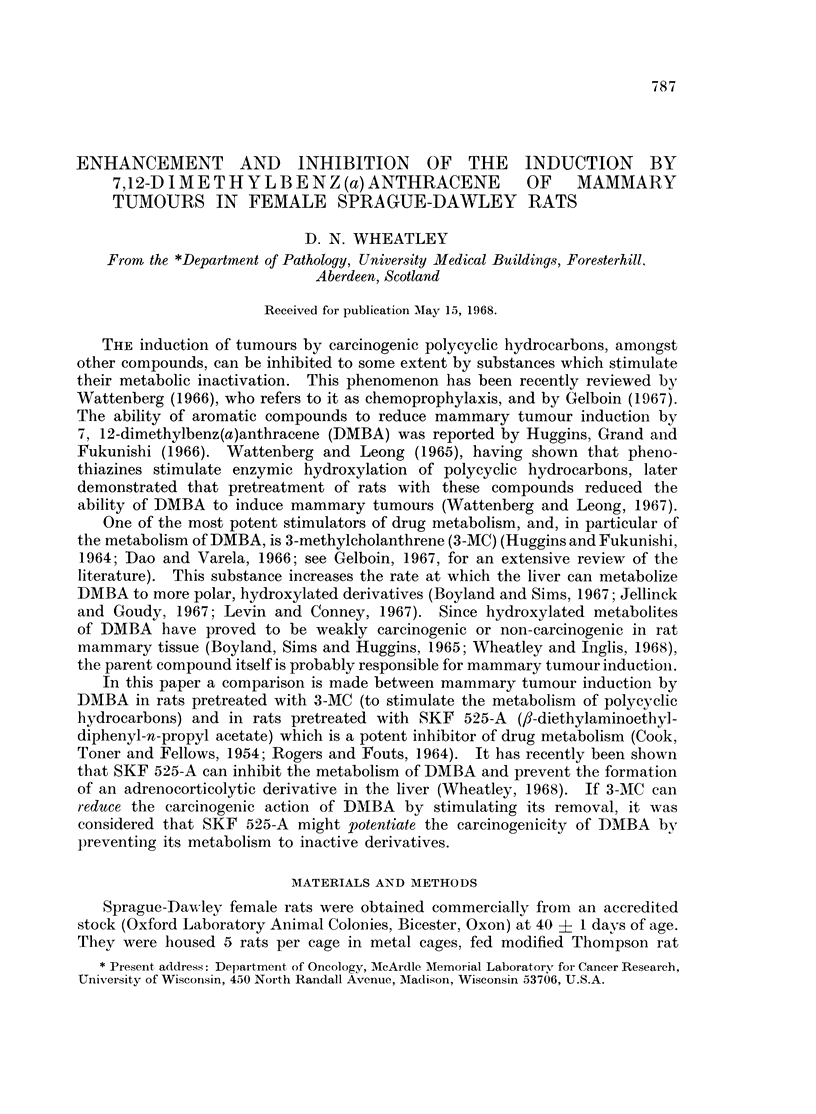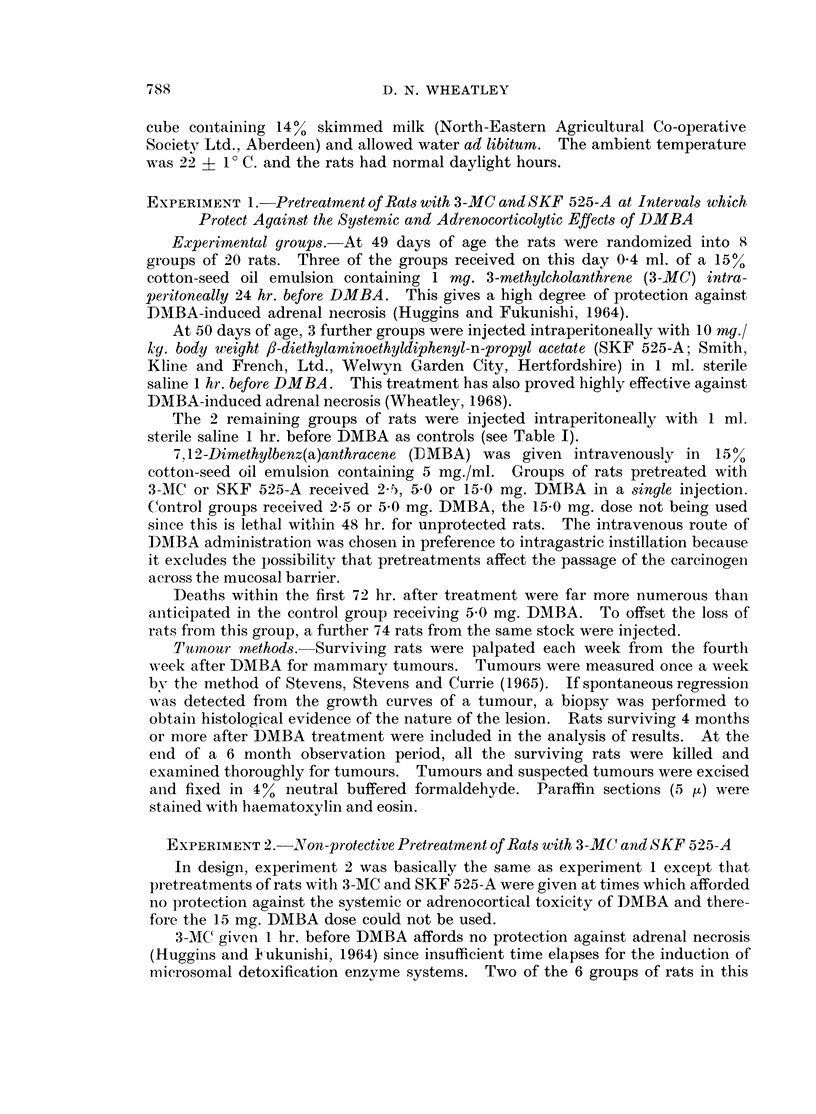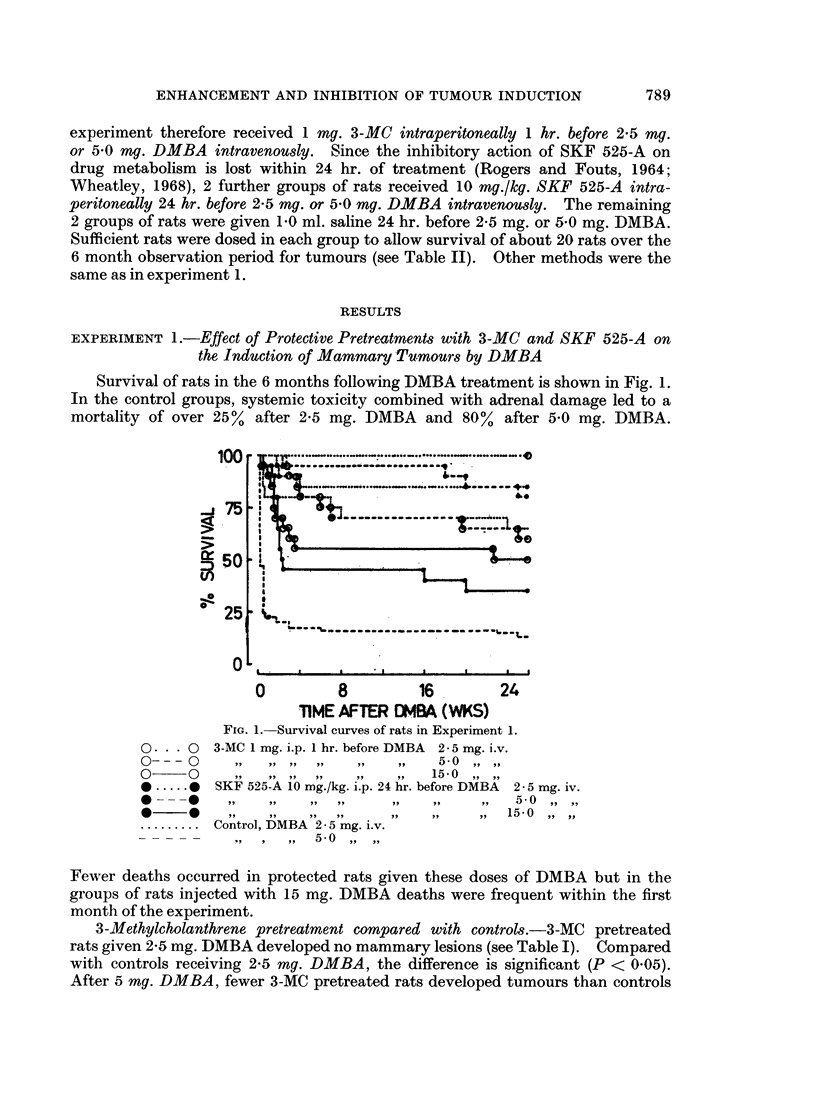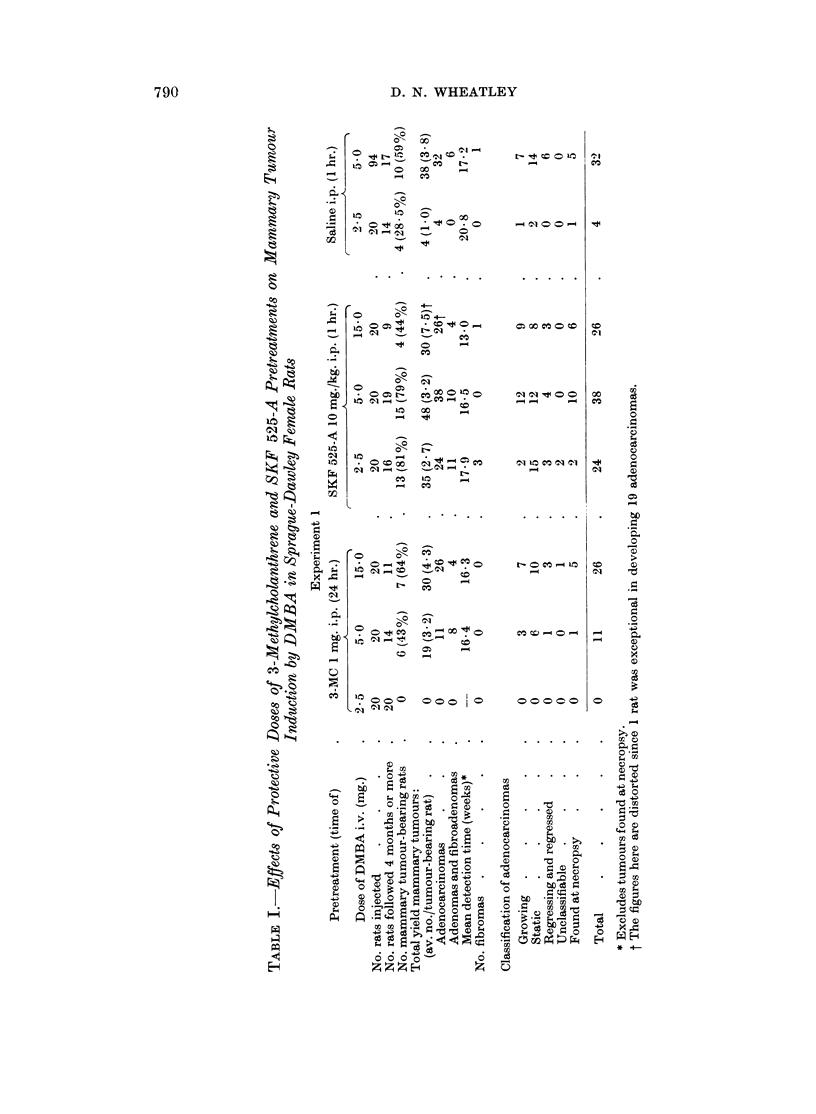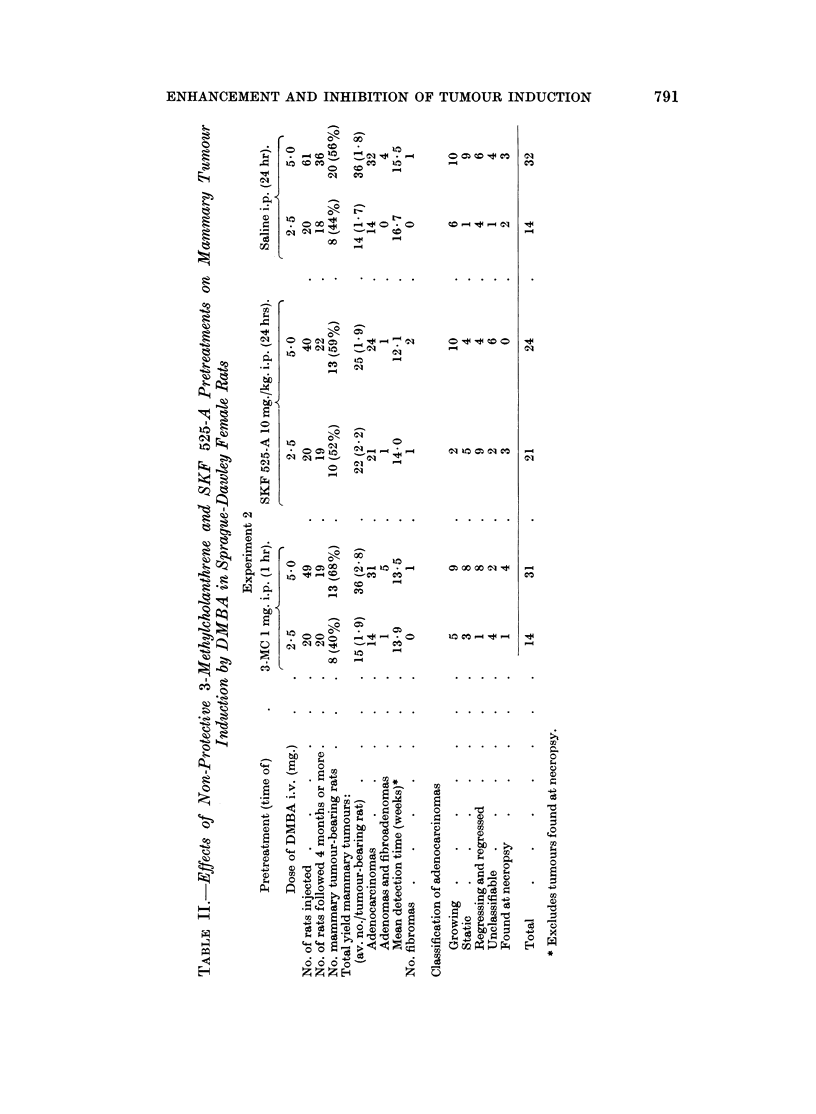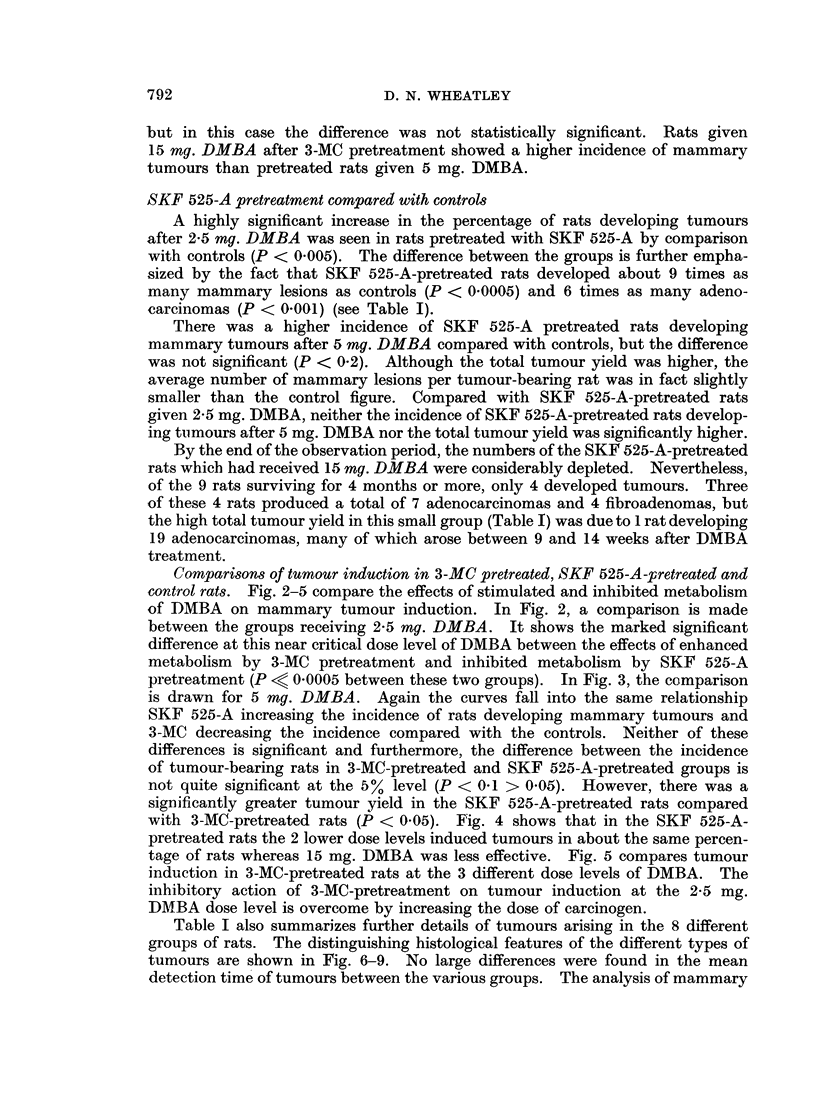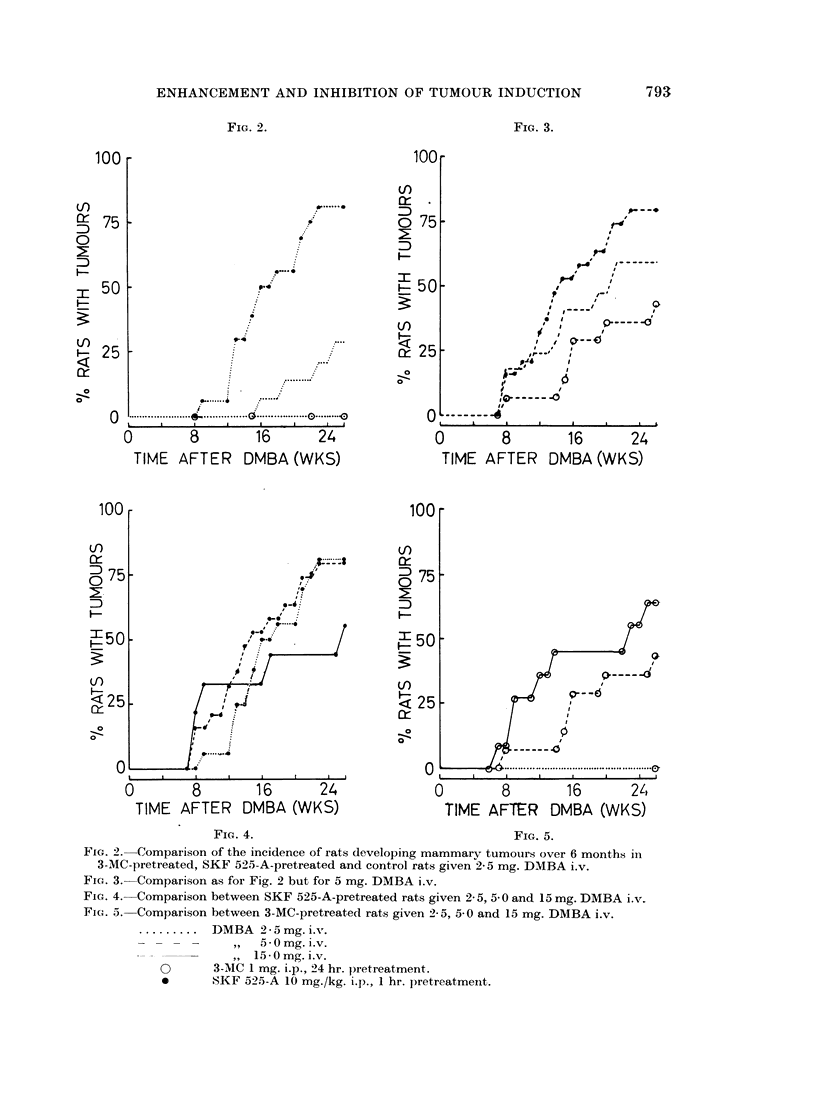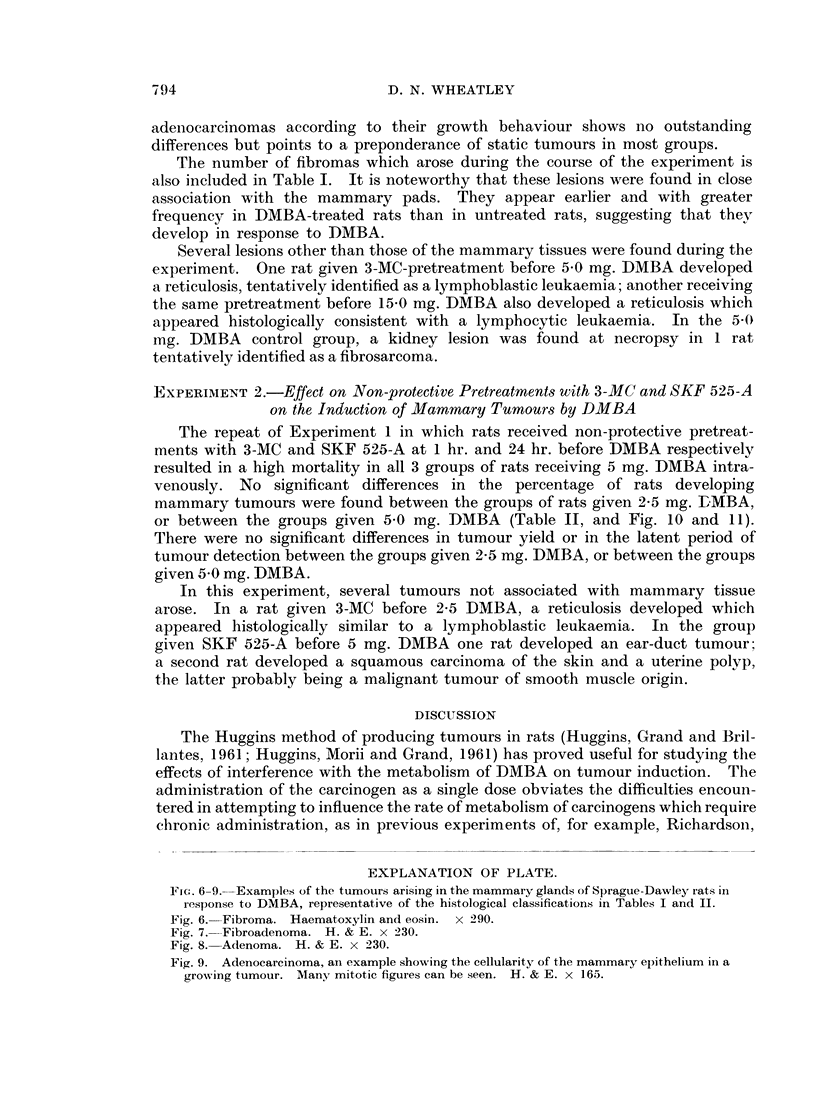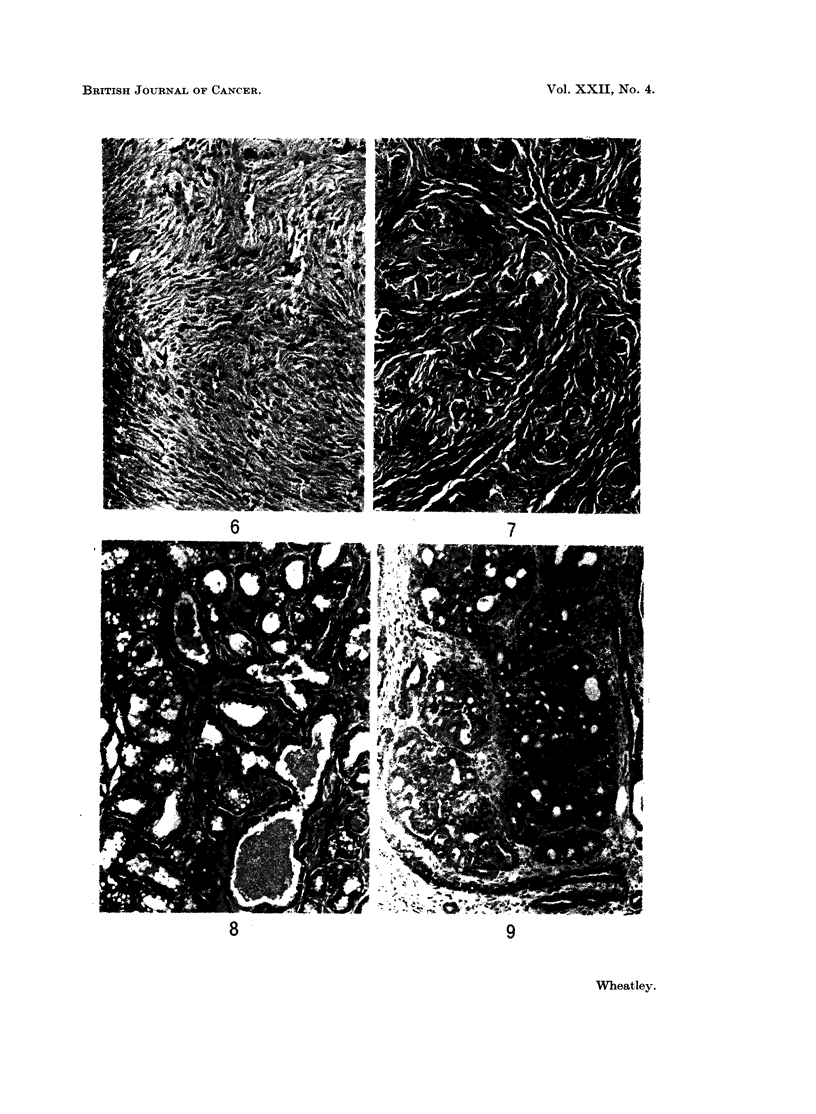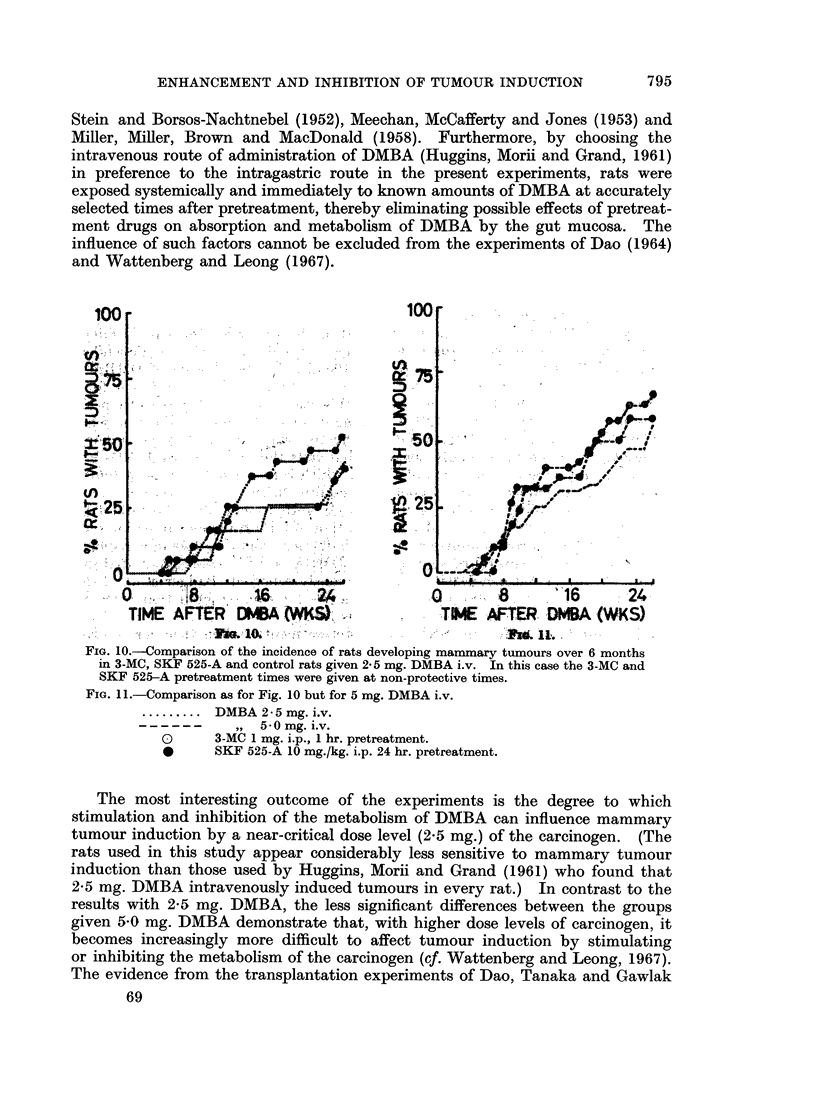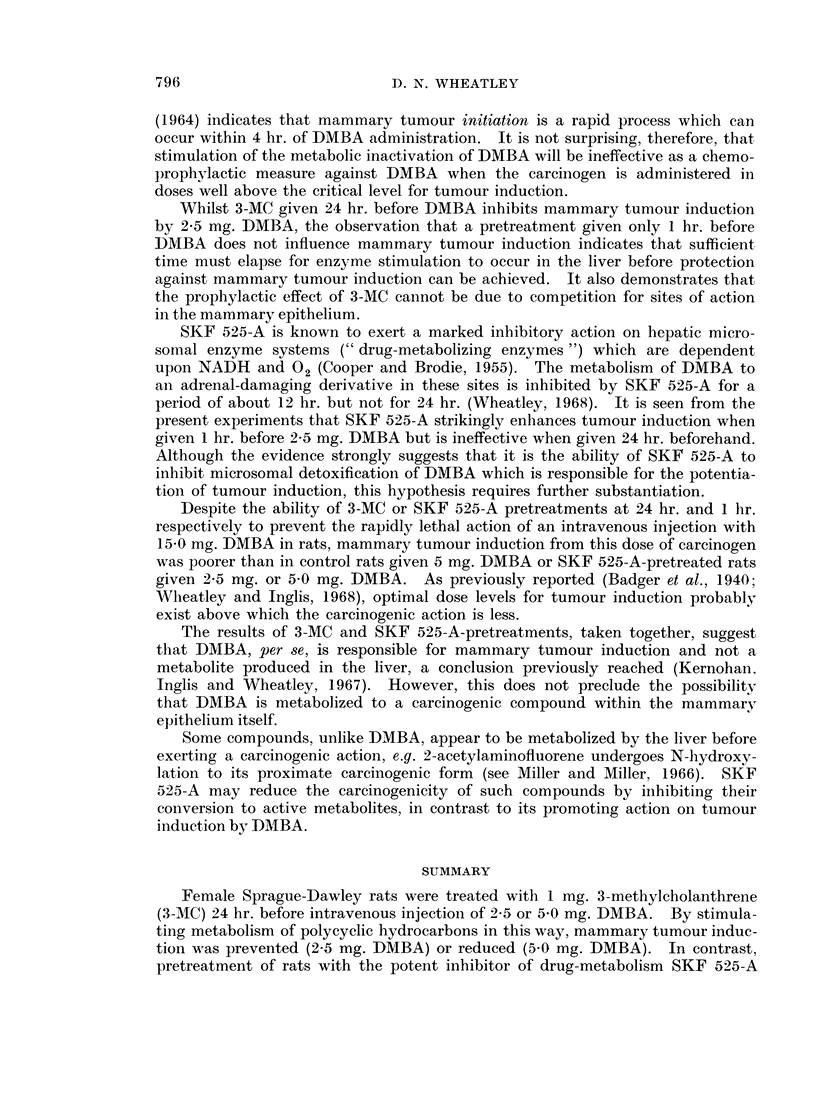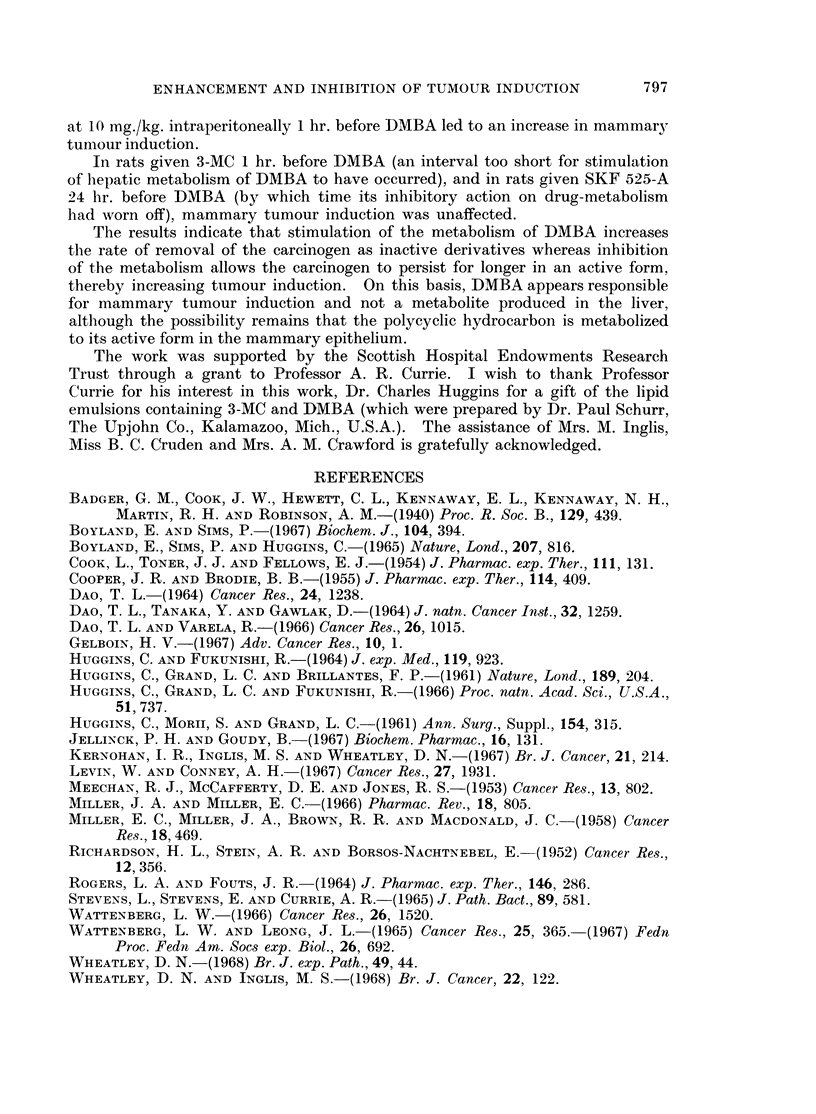# Enhancement and inhibition of the induction by 7,12-dimethylbenz(a)anthracene of mammary tumours in female Sprague-Dawley rats.

**DOI:** 10.1038/bjc.1968.93

**Published:** 1968-12

**Authors:** D. N. Wheatley

## Abstract

**Images:**


### ENHANCEMENT AND INHIBITION OF THE INDUCTION BY 7,12-DIMETHYLBENZ(*a*)ANTHRACENE OF MAMMARY TUMOURS IN FEMALE SPRAGUE-DAWLEY RATS

D. N. WHEATLEY

*From the *Department of Pathology, University Medical Buildings, Foresterhill, Aberdeen, Scotland*

Received for publication May 15, 1968.

THE induction of tumours by carcinogenic polycyclic hydrocarbons, amongst other compounds, can be inhibited to some extent by substances which stimulate their metabolic inactivation. This phenomenon has been recently reviewed by Wattenberg (1966), who refers to it as chemoprophylaxis, and by Gelboin (1967). The ability of aromatic compounds to reduce mammary tumour induction by 7, 12-dimethylbenz(*a*)anthracene (DMBA) was reported by Huggins, Grand and Fukunishi (1966). Wattenberg and Leong (1965), having shown that phenothiazines stimulate enzymic hydroxylation of polycyclic hydrocarbons, later demonstrated that pretreatment of rats with these compounds reduced the ability of DMBA to induce mammary tumours (Wattenberg and Leong, 1967).

One of the most potent stimulators of drug metabolism, and, in particular of the metabolism of DMBA, is 3-methylcholanthrene (3-MC) (Huggins and Fukunishi, 1964; Dao and Varela, 1966; see Gelboin, 1967, for an extensive review of the literature). This substance increases the rate at which the liver can metabolize DMBA to more polar, hydroxylated derivatives (Boyland and Sims, 1967; Jellinck and Goudy, 1967; Levin and Conney, 1967). Since hydroxylated metabolites of DMBA have proved to be weakly carcinogenic or non-carcinogenic in rat mammary tissue (Boyland, Sims and Huggins, 1965; Wheatley and Inglis, 1968), the parent compound itself is probably responsible for mammary tumour induction.

In this paper a comparison is made between mammary tumour induction by DMBA in rats pretreated with 3-MC (to stimulate the metabolism of polycyclic hydrocarbons) and in rats pretreated with SKF 525-A ($\beta$-diethylaminoethyl-diphenyl-*n*-propyl acetate) which is a potent inhibitor of drug metabolism (Cook, Toner and Fellows, 1954; Rogers and Fouts, 1964). It has recently been shown that SKF 525-A can inhibit the metabolism of DMBA and prevent the formation of an adrenocorticolytic derivative in the liver (Wheatley, 1968). If 3-MC can *reduce* the carcinogenic action of DMBA by stimulating its removal, it was considered that SKF 525-A might *potentiate* the carcinogenicity of DMBA by preventing its metabolism to inactive derivatives.

### MATERIALS AND METHODS

Sprague-Dawley female rats were obtained commercially from an accredited stock (Oxford Laboratory Animal Colonies, Bicester, Oxon) at 40 $\pm$ 1 days of age. They were housed 5 rats per cage in metal cages, fed modified Thompson rat

* Present address: Department of Oncology, McArdle Memorial Laboratory for Cancer Research, University of Wisconsin, 450 North Randall Avenue, Madison, Wisconsin 53706, U.S.A.

cube containing 14% skimmed milk (North-Eastern Agricultural Co-operative Society Ltd., Aberdeen) and allowed water *ad libitum.* The ambient temperature was 22 ± 1° C. and the rats had normal daylight hours.

EXPERIMENT 1.—*Pretreatment of Rats with* 3-*MC and SKF* 525-*A at Intervals which Protect Against the Systemic and Adrenocorticolytic Effects of DMBA*

*Experimental groups.*—At 49 days of age the rats were randomized into 8 groups of 20 rats. Three of the groups received on this day 0·4 ml. of a 15% cotton-seed oil emulsion containing 1 *mg.* 3-*methylcholanthrene* (3-*MC*) *intraperitoneally* 24 *hr. before DMBA.* This gives a high degree of protection against DMBA-induced adrenal necrosis (Huggins and Fukunishi, 1964).

At 50 days of age, 3 further groups were injected intraperitoneally with 10 *mg./kg. body weight β-diethylaminoethyldiphenyl*-n-*propyl acetate* (SKF 525-A; Smith, Kline and French, Ltd., Welwyn Garden City, Hertfordshire) in 1 ml. sterile saline 1 *hr. before DMBA.* This treatment has also proved highly effective against DMBA-induced adrenal necrosis (Wheatley, 1968).

The 2 remaining groups of rats were injected intraperitoneally with 1 ml. sterile saline 1 hr. before DMBA as controls (see Table I).

7,12-*Dimethylbenz*(a)*anthracene* (DMBA) was given intravenously in 15% cotton-seed oil emulsion containing 5 mg./ml. Groups of rats pretreated with 3-MC or SKF 525-A received 2·5, 5·0 or 15·0 mg. DMBA in a *single* injection. Control groups received 2·5 or 5·0 mg. DMBA, the 15·0 mg. dose not being used since this is lethal within 48 hr. for unprotected rats. The intravenous route of DMBA administration was chosen in preference to intragastric instillation because it excludes the possibility that pretreatments affect the passage of the carcinogen across the mucosal barrier.

Deaths within the first 72 hr. after treatment were far more numerous than anticipated in the control group receiving 5·0 mg. DMBA. To offset the loss of rats from this group, a further 74 rats from the same stock were injected.

*Tumour methods.*—Surviving rats were palpated each week from the fourth week after DMBA for mammary tumours. Tumours were measured once a week by the method of Stevens, Stevens and Currie (1965). If spontaneous regression was detected from the growth curves of a tumour, a biopsy was performed to obtain histological evidence of the nature of the lesion. Rats surviving 4 months or more after DMBA treatment were included in the analysis of results. At the end of a 6 month observation period, all the surviving rats were killed and examined thoroughly for tumours. Tumours and suspected tumours were excised and fixed in 4% neutral buffered formaldehyde. Paraffin sections (5 $\mu$) were stained with haematoxylin and eosin.

EXPERIMENT 2.—*Non-protective Pretreatment of Rats with* 3-*MC and SKF* 525-*A*

In design, experiment 2 was basically the same as experiment 1 except that pretreatments of rats with 3-MC and SKF 525-A were given at times which afforded no protection against the systemic or adrenocortical toxicity of DMBA and therefore the 15 mg. DMBA dose could not be used.

3-MC given 1 hr. before DMBA affords no protection against adrenal necrosis (Huggins and Fukunishi, 1964) since insufficient time elapses for the induction of microsomal detoxification enzyme systems. Two of the 6 groups of rats in this experiment therefore received 1 *mg. 3-MC intraperitoneally* 1 *hr. before* 2·5 *mg. or* 5·0 *mg. DMBA intravenously.* Since the inhibitory action of SKF 525-A on drug metabolism is lost within 24 hr. of treatment (Rogers and Fouts, 1964; Wheatley, 1968), 2 further groups of rats received 10 *mg./kg. SKF* 525-*A intraperitoneally* 24 *hr. before* 2·5 *mg. or* 5·0 *mg. DMBA intravenously.* The remaining 2 groups of rats were given 1·0 ml. saline 24 hr. before 2·5 mg. or 5·0 mg. DMBA. Sufficient rats were dosed in each group to allow survival of about 20 rats over the 6 month observation period for tumours (see Table II). Other methods were the same as in experiment 1.

### RESULTS

EXPERIMENT 1.—*Effect of Protective Pretreatments with 3-MC and SKF 525-A on the Induction of Mammary Tumours by DMBA*

Survival of rats in the 6 months following DMBA treatment is shown in Fig. 1. In the control groups, systemic toxicity combined with adrenal damage led to a mortality of over 25% after 2·5 mg. DMBA and 80% after 5·0 mg. DMBA.

FIG. 1.—Survival curves of rats in Experiment 1.

○ . . . ○ 3-MC 1 mg. i.p. 1 hr. before DMBA 2·5 mg. i.v.

○ – – – ○ ,, ,, ,, ,, ,, ,, 5·0 ,, ,,

○——○ ,, ,, ,, ,, ,, ,, 15·0 ,, ,,

● . . . . . ● SKF 525-A 10 mg./kg. i.p. 24 hr. before DMBA 2·5 mg. iv.

● – – – ● ,, ,, ,, ,, ,, ,, ,, 5·0 ,, ,,

●——● ,, ,, ,, ,, ,, ,, ,, 15·0 ,, ,,

. . . . . . . . . Control, DMBA 2·5 mg. i.v.

– – – – – – ,, , ,, 5·0 ,, ,,

Fewer deaths occurred in protected rats given these doses of DMBA but in the groups of rats injected with 15 mg. DMBA deaths were frequent within the first month of the experiment.

3-*Methylcholanthrene pretreatment compared with controls.*—3-MC pretreated rats given 2·5 mg. DMBA developed no mammary lesions (see Table I). Compared with controls receiving 2·5 *mg. DMBA*, the difference is significant ($P < 0{\cdot}05$). After 5 *mg. DMBA*, fewer 3-MC pretreated rats developed tumours than controls

TABLE I.—*Effects of Protective Doses of 3-Methylcholanthrene and SKF 525-A Pretreatments on Mammary Tumour Induction by DMBA in Sprague-Dawley Female Rats*

Experiment 1

| Pretreatment (time of) | 3-MC 1 mg. i.p. (24 hr.) | | | SKF 525-A 10 mg./kg. i.p. (1 hr.) | | | Saline i.p. (1 hr.) | |
|---|---|---|---|---|---|---|---|---|
| Dose of DMBA i.v. (mg.) | 2·5 | 5·0 | 15·0 | 2·5 | 5·0 | 15·0 | 2·5 | 5·0 |
| No. rats injected | 20 | 20 | 20 | 20 | 20 | 20 | 20 | 94 |
| No. rats followed 4 months or more | 20 | 14 | 11 | 16 | 19 | 9 | 14 | 17 |
| No. mammary tumour-bearing rats | 0 | 6 (43%) | 7 (64%) | 13 (81%) | 15 (79%) | 4 (44%) | 4 (28·5%) | 10 (59%) |
| Total yield mammary tumours: | | | | | | | | |
| (av. no./tumour-bearing rat) | 0 | 19 (3·2) | 30 (4·3) | 35 (2·7) | 48 (3·2) | 30 (7·5)† | 4 (1·0) | 38 (3·8) |
| Adenocarcinomas | 0 | 11 | 26 | 24 | 38 | 26† | 4 | 32 |
| Adenomas and fibroadenomas | 0 | 8 | 4 | 11 | 10 | 4 | 0 | 6 |
| Mean detection time (weeks)* | — | 16·4 | 16·3 | 17·9 | 16·5 | 13·0 | 20·8 | 17·2 |
| No. fibromas | 0 | 0 | 0 | 3 | 0 | 1 | 0 | 1 |
| Classification of adenocarcinomas | | | | | | | | |
| Growing | 0 | 3 | 7 | 2 | 12 | 9 | 1 | 7 |
| Static | 0 | 6 | 10 | 15 | 12 | 8 | 2 | 14 |
| Regressing and regressed | 0 | 1 | 3 | 3 | 4 | 3 | 0 | 6 |
| Unclassifiable | 0 | 0 | 1 | 2 | 0 | 0 | 0 | 0 |
| Found at necropsy | 0 | 1 | 5 | 2 | 10 | 6 | 1 | 5 |
| Total | 0 | 11 | 26 | 24 | 38 | 26 | 4 | 32 |

* Excludes tumours found at necropsy.

† The figures here are distorted since 1 rat was exceptional in developing 19 adenocarcinomas.

TABLE II.—*Effects of Non-Protective 3-Methylcholanthrene and SKF 525-A Pretreatments on Mammary Tumour Induction by DMBA in Sprague-Dawley Female Rats*

| Experiment 2 | | | | | | |
|---|---|---|---|---|---|---|
| Pretreatment (time of) | 3-MC 1 mg. i.p. (1 hr). | | SKF 525-A 10 mg./kg. i.p. (24 hrs). | | Saline i.p. (24 hr). | |
| Dose of DMBA i.v. (mg.) | 2·5 | 5·0 | 2·5 | 5·0 | 2·5 | 5·0 |
| No. of rats injected | 20 | 49 | 20 | 40 | 20 | 61 |
| No. of rats followed 4 months or more | 20 | 19 | 19 | 22 | 18 | 36 |
| No. mammary tumour-bearing rats | 8 (40%) | 13 (68%) | 10 (52%) | 13 (59%) | 8 (44%) | 20 (56%) |
| Total yield mammary tumours: | | | | | | |
| (av. no./tumour-bearing rat) | 15 (1·9) | 36 (2·8) | 22 (2·2) | 25 (1·9) | 14 (1·7) | 36 (1·8) |
| Adenocarcinomas | 14 | 31 | 21 | 24 | 14 | 32 |
| Adenomas and fibroadenomas | 1 | 5 | 1 | 1 | 0 | 4 |
| Mean detection time (weeks)* | 13·9 | 13·5 | 14·0 | 12·1 | 16·7 | 15·5 |
| No. fibromas | 0 | 1 | 1 | 2 | 0 | 1 |
| Classification of adenocarcinomas | | | | | | |
| Growing | 5 | 9 | 2 | 10 | 6 | 10 |
| Static | 3 | 8 | 5 | 4 | 1 | 9 |
| Regressing and regressed | 1 | 8 | 9 | 4 | 4 | 6 |
| Unclassifiable | 4 | 2 | 2 | 6 | 1 | 4 |
| Found at necropsy | 1 | 4 | 3 | 0 | 2 | 3 |
| Total | 14 | 31 | 21 | 24 | 14 | 32 |

* Excludes tumours found at necropsy.

but in this case the difference was not statistically significant. Rats given 15 *mg. DMBA* after 3-MC pretreatment showed a higher incidence of mammary tumours than pretreated rats given 5 mg. DMBA.

*SKF 525-A pretreatment compared with controls*

A highly significant increase in the percentage of rats developing tumours after 2·5 *mg. DMBA* was seen in rats pretreated with SKF 525-A by comparison with controls ($P < 0{\cdot}005$). The difference between the groups is further emphasized by the fact that SKF 525-A-pretreated rats developed about 9 times as many mammary lesions as controls ($P < 0{\cdot}0005$) and 6 times as many adenocarcinomas ($P < 0{\cdot}001$) (see Table I).

There was a higher incidence of SKF 525-A pretreated rats developing mammary tumours after 5 *mg. DMBA* compared with controls, but the difference was not significant ($P < 0{\cdot}2$). Although the total tumour yield was higher, the average number of mammary lesions per tumour-bearing rat was in fact slightly smaller than the control figure. Compared with SKF 525-A-pretreated rats given 2·5 mg. DMBA, neither the incidence of SKF 525-A-pretreated rats developing tumours after 5 mg. DMBA nor the total tumour yield was significantly higher.

By the end of the observation period, the numbers of the SKF 525-A-pretreated rats which had received 15 *mg. DMBA* were considerably depleted. Nevertheless, of the 9 rats surviving for 4 months or more, only 4 developed tumours. Three of these 4 rats produced a total of 7 adenocarcinomas and 4 fibroadenomas, but the high total tumour yield in this small group (Table I) was due to 1 rat developing 19 adenocarcinomas, many of which arose between 9 and 14 weeks after DMBA treatment.

*Comparisons of tumour induction in 3-MC pretreated, SKF 525-A-pretreated and control rats.* Fig. 2–5 compare the effects of stimulated and inhibited metabolism of DMBA on mammary tumour induction. In Fig. 2, a comparison is made between the groups receiving 2·5 *mg. DMBA*. It shows the marked significant difference at this near critical dose level of DMBA between the effects of enhanced metabolism by 3-MC pretreatment and inhibited metabolism by SKF 525-A pretreatment ($P \ll 0{\cdot}0005$ between these two groups). In Fig. 3, the comparison is drawn for 5 *mg. DMBA*. Again the curves fall into the same relationship SKF 525-A increasing the incidence of rats developing mammary tumours and 3-MC decreasing the incidence compared with the controls. Neither of these differences is significant and furthermore, the difference between the incidence of tumour-bearing rats in 3-MC-pretreated and SKF 525-A-pretreated groups is not quite significant at the 5% level ($P < 0{\cdot}1 > 0{\cdot}05$). However, there was a significantly greater tumour yield in the SKF 525-A-pretreated rats compared with 3-MC-pretreated rats ($P < 0{\cdot}05$). Fig. 4 shows that in the SKF 525-A-pretreated rats the 2 lower dose levels induced tumours in about the same percentage of rats whereas 15 mg. DMBA was less effective. Fig. 5 compares tumour induction in 3-MC-pretreated rats at the 3 different dose levels of DMBA. The inhibitory action of 3-MC-pretreatment on tumour induction at the 2·5 mg. DMBA dose level is overcome by increasing the dose of carcinogen.

Table I also summarizes further details of tumours arising in the 8 different groups of rats. The distinguishing histological features of the different types of tumours are shown in Fig. 6–9. No large differences were found in the mean detection time of tumours between the various groups. The analysis of mammary

Fig. 2.

Fig. 3.

Fig. 4.

Fig. 5.

Fig. 2.—Comparison of the incidence of rats developing mammary tumours over 6 months in 3-MC-pretreated, SKF 525-A-pretreated and control rats given 2·5 mg. DMBA i.v.

Fig. 3.—Comparison as for Fig. 2 but for 5 mg. DMBA i.v.

Fig. 4.—Comparison between SKF 525-A-pretreated rats given 2·5, 5·0 and 15 mg. DMBA i.v.

Fig. 5.—Comparison between 3-MC-pretreated rats given 2·5, 5·0 and 15 mg. DMBA i.v.

| | |
|---|---|
| ......... | DMBA 2·5 mg. i.v. |
| – – – – | ,, 5·0 mg. i.v. |
| —— | ,, 15·0 mg. i.v. |
| ○ | 3-MC 1 mg. i.p., 24 hr. pretreatment. |
| ● | SKF 525-A 10 mg./kg. i.p., 1 hr. pretreatment. |

adenocarcinomas according to their growth behaviour shows no outstanding differences but points to a preponderance of static tumours in most groups.

The number of fibromas which arose during the course of the experiment is also included in Table I. It is noteworthy that these lesions were found in close association with the mammary pads. They appear earlier and with greater frequency in DMBA-treated rats than in untreated rats, suggesting that they develop in response to DMBA.

Several lesions other than those of the mammary tissues were found during the experiment. One rat given 3-MC-pretreatment before 5·0 mg. DMBA developed a reticulosis, tentatively identified as a lymphoblastic leukaemia; another receiving the same pretreatment before 15·0 mg. DMBA also developed a reticulosis which appeared histologically consistent with a lymphocytic leukaemia. In the 5·0 mg. DMBA control group, a kidney lesion was found at necropsy in 1 rat tentatively identified as a fibrosarcoma.

EXPERIMENT 2.—*Effect on Non-protective Pretreatments with 3-MC and SKF 525-A on the Induction of Mammary Tumours by DMBA*

The repeat of Experiment 1 in which rats received non-protective pretreatments with 3-MC and SKF 525-A at 1 hr. and 24 hr. before DMBA respectively resulted in a high mortality in all 3 groups of rats receiving 5 mg. DMBA intravenously. No significant differences in the percentage of rats developing mammary tumours were found between the groups of rats given 2·5 mg. DMBA, or between the groups given 5·0 mg. DMBA (Table II, and Fig. 10 and 11). There were no significant differences in tumour yield or in the latent period of tumour detection between the groups given 2·5 mg. DMBA, or between the groups given 5·0 mg. DMBA.

In this experiment, several tumours not associated with mammary tissue arose. In a rat given 3-MC before 2·5 DMBA, a reticulosis developed which appeared histologically similar to a lymphoblastic leukaemia. In the group given SKF 525-A before 5 mg. DMBA one rat developed an ear-duct tumour; a second rat developed a squamous carcinoma of the skin and a uterine polyp, the latter probably being a malignant tumour of smooth muscle origin.

### DISCUSSION

The Huggins method of producing tumours in rats (Huggins, Grand and Brillantes, 1961; Huggins, Morii and Grand, 1961) has proved useful for studying the effects of interference with the metabolism of DMBA on tumour induction. The administration of the carcinogen as a single dose obviates the difficulties encountered in attempting to influence the rate of metabolism of carcinogens which require chronic administration, as in previous experiments of, for example, Richardson,

---

EXPLANATION OF PLATE.

FIG. 6–9.—Examples of the tumours arising in the mammary glands of Sprague-Dawley rats in response to DMBA, representative of the histological classifications in Tables I and II.

Fig. 6.—Fibroma. Haematoxylin and eosin. × 290.

Fig. 7.—Fibroadenoma. H. & E. × 230.

Fig. 8.—Adenoma. H. & E. × 230.

Fig. 9. Adenocarcinoma, an example showing the cellularity of the mammary epithelium in a growing tumour. Many mitotic figures can be seen. H. & E. × 165.

6 7 8 9

Wheatley.

Stein and Borsos-Nachtnebel (1952), Meechan, McCafferty and Jones (1953) and Miller, Miller, Brown and MacDonald (1958). Furthermore, by choosing the intravenous route of administration of DMBA (Huggins, Morii and Grand, 1961) in preference to the intragastric route in the present experiments, rats were exposed systemically and immediately to known amounts of DMBA at accurately selected times after pretreatment, thereby eliminating possible effects of pretreatment drugs on absorption and metabolism of DMBA by the gut mucosa. The influence of such factors cannot be excluded from the experiments of Dao (1964) and Wattenberg and Leong (1967).

FIG. 10.

FIG. 11.

FIG. 10.—Comparison of the incidence of rats developing mammary tumours over 6 months in 3-MC, SKF 525-A and control rats given 2·5 mg. DMBA i.v. In this case the 3-MC and SKF 525–A pretreatment times were given at non-protective times.

FIG. 11.—Comparison as for Fig. 10 but for 5 mg. DMBA i.v.

......... DMBA 2·5 mg. i.v.
------ „ 5·0 mg. i.v.
○ 3-MC 1 mg. i.p., 1 hr. pretreatment.
● SKF 525-A 10 mg./kg. i.p. 24 hr. pretreatment.

The most interesting outcome of the experiments is the degree to which stimulation and inhibition of the metabolism of DMBA can influence mammary tumour induction by a near-critical dose level (2·5 mg.) of the carcinogen. (The rats used in this study appear considerably less sensitive to mammary tumour induction than those used by Huggins, Morii and Grand (1961) who found that 2·5 mg. DMBA intravenously induced tumours in every rat.) In contrast to the results with 2·5 mg. DMBA, the less significant differences between the groups given 5·0 mg. DMBA demonstrate that, with higher dose levels of carcinogen, it becomes increasingly more difficult to affect tumour induction by stimulating or inhibiting the metabolism of the carcinogen (*cf.* Wattenberg and Leong, 1967). The evidence from the transplantation experiments of Dao, Tanaka and Gawlak

(1964) indicates that mammary tumour *initiation* is a rapid process which can occur within 4 hr. of DMBA administration. It is not surprising, therefore, that stimulation of the metabolic inactivation of DMBA will be ineffective as a chemoprophylactic measure against DMBA when the carcinogen is administered in doses well above the critical level for tumour induction.

Whilst 3-MC given 24 hr. before DMBA inhibits mammary tumour induction by 2·5 mg. DMBA, the observation that a pretreatment given only 1 hr. before DMBA does not influence mammary tumour induction indicates that sufficient time must elapse for enzyme stimulation to occur in the liver before protection against mammary tumour induction can be achieved. It also demonstrates that the prophylactic effect of 3-MC cannot be due to competition for sites of action in the mammary epithelium.

SKF 525-A is known to exert a marked inhibitory action on hepatic microsomal enzyme systems (" drug-metabolizing enzymes ") which are dependent upon NADH and $O_2$ (Cooper and Brodie, 1955). The metabolism of DMBA to an adrenal-damaging derivative in these sites is inhibited by SKF 525-A for a period of about 12 hr. but not for 24 hr. (Wheatley, 1968). It is seen from the present experiments that SKF 525-A strikingly enhances tumour induction when given 1 hr. before 2·5 mg. DMBA but is ineffective when given 24 hr. beforehand. Although the evidence strongly suggests that it is the ability of SKF 525-A to inhibit microsomal detoxification of DMBA which is responsible for the potentiation of tumour induction, this hypothesis requires further substantiation.

Despite the ability of 3-MC or SKF 525-A pretreatments at 24 hr. and 1 hr. respectively to prevent the rapidly lethal action of an intravenous injection with 15·0 mg. DMBA in rats, mammary tumour induction from this dose of carcinogen was poorer than in control rats given 5 mg. DMBA or SKF 525-A-pretreated rats given 2·5 mg. or 5·0 mg. DMBA. As previously reported (Badger *et al.*, 1940; Wheatley and Inglis, 1968), optimal dose levels for tumour induction probably exist above which the carcinogenic action is less.

The results of 3-MC and SKF 525-A-pretreatments, taken together, suggest that DMBA, *per se*, is responsible for mammary tumour induction and not a metabolite produced in the liver, a conclusion previously reached (Kernohan, Inglis and Wheatley, 1967). However, this does not preclude the possibility that DMBA is metabolized to a carcinogenic compound within the mammary epithelium itself.

Some compounds, unlike DMBA, appear to be metabolized by the liver before exerting a carcinogenic action, *e.g.* 2-acetylaminofluorene undergoes N-hydroxylation to its proximate carcinogenic form (see Miller and Miller, 1966). SKF 525-A may reduce the carcinogenicity of such compounds by inhibiting their conversion to active metabolites, in contrast to its promoting action on tumour induction by DMBA.

### SUMMARY

Female Sprague-Dawley rats were treated with 1 mg. 3-methylcholanthrene (3-MC) 24 hr. before intravenous injection of 2·5 or 5·0 mg. DMBA. By stimulating metabolism of polycyclic hydrocarbons in this way, mammary tumour induction was prevented (2·5 mg. DMBA) or reduced (5·0 mg. DMBA). In contrast, pretreatment of rats with the potent inhibitor of drug-metabolism SKF 525-A

at 10 mg./kg. intraperitoneally 1 hr. before DMBA led to an increase in mammary tumour induction.

In rats given 3-MC 1 hr. before DMBA (an interval too short for stimulation of hepatic metabolism of DMBA to have occurred), and in rats given SKF 525-A 24 hr. before DMBA (by which time its inhibitory action on drug-metabolism had worn off), mammary tumour induction was unaffected.

The results indicate that stimulation of the metabolism of DMBA increases the rate of removal of the carcinogen as inactive derivatives whereas inhibition of the metabolism allows the carcinogen to persist for longer in an active form, thereby increasing tumour induction. On this basis, DMBA appears responsible for mammary tumour induction and not a metabolite produced in the liver, although the possibility remains that the polycyclic hydrocarbon is metabolized to its active form in the mammary epithelium.

The work was supported by the Scottish Hospital Endowments Research Trust through a grant to Professor A. R. Currie. I wish to thank Professor Currie for his interest in this work, Dr. Charles Huggins for a gift of the lipid emulsions containing 3-MC and DMBA (which were prepared by Dr. Paul Schurr, The Upjohn Co., Kalamazoo, Mich., U.S.A.). The assistance of Mrs. M. Inglis, Miss B. C. Cruden and Mrs. A. M. Crawford is gratefully acknowledged.

### REFERENCES

Badger, G. M., Cook, J. W., Hewett, C. L., Kennaway, E. L., Kennaway, N. H., Martin, R. H. and Robinson, A. M.—(1940) *Proc. R. Soc.* B., **129**, 439.

Boyland, E. and Sims, P.—(1967) *Biochem. J.*, **104**, 394.

Boyland, E., Sims, P. and Huggins, C.—(1965) *Nature, Lond.*, **207**, 816.

Cook, L., Toner, J. J. and Fellows, E. J.—(1954) *J. Pharmac. exp. Ther.*, **111**, 131.

Cooper, J. R. and Brodie, B. B.—(1955) *J. Pharmac. exp. Ther.*, **114**, 409.

Dao, T. L.—(1964) *Cancer Res.*, **24**, 1238.

Dao, T. L., Tanaka, Y. and Gawlak, D.—(1964) *J. natn. Cancer Inst.*, **32**, 1259.

Dao, T. L. and Varela, R.—(1966) *Cancer Res.*, **26**, 1015.

Gelboin, H. V.—(1967) *Adv. Cancer Res.*, **10**, 1.

Huggins, C. and Fukunishi, R.—(1964) *J. exp. Med.*, **119**, 923.

Huggins, C., Grand, L. C. and Brillantes, F. P.—(1961) *Nature, Lond.*, **189**, 204.

Huggins, C., Grand, L. C. and Fukunishi, R.—(1966) *Proc. natn. Acad. Sci., U.S.A.*, **51**, 737.

Huggins, C., Morii, S. and Grand, L. C.—(1961) *Ann. Surg.*, Suppl., **154**, 315.

Jellinck, P. H. and Goudy, B.—(1967) *Biochem. Pharmac.*, **16**, 131.

Kernohan, I. R., Inglis, M. S. and Wheatley, D. N.—(1967) *Br. J. Cancer*, **21**, 214.

Levin, W. and Conney, A. H.—(1967) *Cancer Res.*, **27**, 1931.

Meechan, R. J., McCafferty, D. E. and Jones, R. S.—(1953) *Cancer Res.*, **13**, 802.

Miller, J. A. and Miller, E. C.—(1966) *Pharmac. Rev.*, **18**, 805.

Miller, E. C., Miller, J. A., Brown, R. R. and Macdonald, J. C.—(1958) *Cancer Res.*, **18**, 469.

Richardson, H. L., Stein, A. R. and Borsos-Nachtnebel, E.—(1952) *Cancer Res.*, **12**, 356.

Rogers, L. A. and Fouts, J. R.—(1964) *J. Pharmac. exp. Ther.*, **146**, 286.

Stevens, L., Stevens, E. and Currie, A. R.—(1965) *J. Path. Bact.*, **89**, 581.

Wattenberg, L. W.—(1966) *Cancer Res.*, **26**, 1520.

Wattenberg, L. W. and Leong, J. L.—(1965) *Cancer Res.*, **25**, 365.—(1967) *Fedn Proc. Fedn Am. Socs exp. Biol.*, **26**, 692.

Wheatley, D. N.—(1968) *Br. J. exp. Path.*, **49**, 44.

Wheatley, D. N. and Inglis, M. S.—(1968) *Br. J. Cancer*, **22**, 122.